# Association of *TP53* rs1042522 C>G Polymorphism with Glioma Risk in Chinese Children

**DOI:** 10.1155/2022/2712808

**Published:** 2022-08-13

**Authors:** Fan Liao, Li Yuan, Jinhong Zhu, Wei Chen, Yemu Zhao, Jing He

**Affiliations:** ^1^School of Medicine, South China University of Technology, Guangzhou, 510006 Guangdong, China; ^2^Department of Pediatric Surgery, Guangzhou Institute of Pediatrics, Guangdong Provincial Key Laboratory of Research in Structural Birth Defect Disease, Guangdong Provincial Clinical Research Center for Child Health, Guangzhou Women and Children's Medical Center, Guangzhou Medical University, Guangzhou, 510623 Guangdong, China; ^3^Department of Pathology, Guangzhou Women and Children's Medical Center, Guangzhou Medical University, Guangzhou, 510623 Guangdong, China; ^4^Department of Clinical Laboratory, Biobank, Harbin Medical University Cancer Hospital, Harbin, 150040 Heilongjiang, China

## Abstract

Glioma is the most common intracranial malignancy. *TP53* is a crucial tumor suppressor gene that plays an essential regulatory role in cell growth, apoptosis, and DNA repair. The *TP53* rs1042522 C>G polymorphism has been reported to be strongly associated with various tumor risks. To assess the *TP53* rs1042522 C>G polymorphism with glioma risk in Chinese children, we determined the genotypes of the *TP53* rs1042522 C>G polymorphism in 171 glioma patients and 228 cancer-free controls by Taqman assay. We assessed the association of the polymorphism with glioma risk using odds ratio (OR) and 95% confidence interval (CI) generated by logistic regression models. We also performed stratified analyses by age, gender, tumor subtypes, and clinical stages, but no significant association was detected between *TP53* rs1042522 C>G polymorphism and childhood glioma risk. These results suggest that the *TP53* rs1042522 C>G polymorphism is not associated with glioma risk in Chinese children. Subsequent studies with a larger sample size are needed to validate our results.

## 1. Introduction

Glioma is a highly heterogeneous tumor and is the most commonly diagnosed primary brain tumor, accounting for 38%~52% of all neurological tumors and 80% of central nervous system (CNS) malignancies. The annual incidence of glioma ranges from 4.6 to 5.7 per 100,000 worldwide, and its incidence has been increasing significantly in recent years [1]. According to the latest cancer statistics report [2], approximately 23,890 brain and other neurological tumor cases were diagnosed in the United States in 2020, of which 18,020 patients have died. The primary treatment for glioma is surgical resection followed by radiotherapy and chemotherapy. Still, the available treatments are not effective against glioma. The prognosis is poor. For instance, the 2-year survival rate for patients with glioblastoma multiforme (GBM), the most malignant form of glioma, is approximately 26.5% with a median survival of only 14.6 months [3]. Glioma is a particularly devastating malignancy. Even though it is extensively studied, the etiology is poorly understood. No defined environmental or lifestyle factors are associated with glioma risk, except for very few cases of exposure to ionizing radiation [4].

Glioma is thought to mainly arise from genetic variants or mutations in critical genes, especially activation of oncogenes such as *EGFR* and inactivation of tumor suppressor genes (e.g., *TP53* and *P16*). Tumor suppressor gene *TP53* is a regulator of cell growth, apoptosis, and DNA repair, and activated *TP53* acts as a transcriptional factor. Its target genes include the cell cycle (*CDKN1A*), apoptosis (*PUMA*), and DNA repair (*14-3-3σ* and *XPC*) [5]. *TP53* is located on chromosome 17p13 [6], with a mutation rate of approximately 25% to 40% in all human malignancies, making it one of the genes mutated most frequently in human cancers [5]. *TP53* plays a vital role in the direct regulation of the tumor cell cycle, apoptosis, and DNA repair or induction of expression of downstream targets. Activation of *TP53*-dependent pathways induced by internal or external cellular stress signals affects cancer cell genesis, progression, and metastasis and prevents the proliferation of damaged cells with oncogenic potential. In addition, as transcription factors, a variety of genes can be activated by *TP53* to promote these specific processes associated with tumor suppression [7]. Significant mutations in *TP53* are present in cells of several cancer types, such as ovarian plasmacytoid cystic adenocarcinoma (95%), small cell lung cancer (90%), carcinoma with characteristic lesions of esophageal squamous cell carcinoma (84%), pancreatic ductal adenocarcinoma (80%), and lung squamous cell carcinoma (79%) [8–11]. Patients with*TP53* mutations in some tumors were found to have a generally worse prognosis than those without *TP53* mutations [12], and mutated *TP53* is a recognized marker for poor prognosis and chemoresistant disease in some tumors [13]. Abnormal expression of *TP53* accelerates the development and progression of various tumors, including glioblastoma. It has also been shown that *TP53* is likely to be mutated across all glioma grades and that the mutation rate is significantly higher in high-grade gliomas compared to low-grade gliomas [14]. Recent genome-wide association studies (GWASs) have illustrated the genetic impact of single-nucleotide polymorphisms (SNPs) at multiple independent loci on glioma risk [15–18]. In a GWAS of over 12,000 glioma patients, 25 SNPs were strongly associated with the risk of glioma development in adults [19]. In another study containing more than 1600 glioma patients, these 25 SNPs were associated with related molecular subtypes such as *IDH* mutations, *TERT* promoter mutations, and *1p19q* codeletions. Of these 25 SNPs, 11 were associated with the risk of glioblastoma, 19 with the risk of astrocytoma and oligodendroglioma, and 5 of these SNPs were associated with the risk of all three glioma types [20]. These data suggest the important contribution of SNPs to glioma susceptibility. Due to the importance of *P53* in tumor suppression, SNPs that alter *P53* function may affect cancer risk and progression. A polymorphism at codon 72 of the *TP53* tumor suppressor gene (rs1042522 C>G) is associated with the risk of several human tumors [21]. However, its association with the risk of glioma in children has rarely been reported. Therefore, we analyzed the association of the *TP53* rs1042522 C>G polymorphism with glioma risk in 171 glioma patients and 228 cancer-free controls.

## 2. Materials and Methods

### 2.1. Study Subjects

We recruited 171 glioma patients and 228 cancer-free controls from China to participate in the study (Table S1) [22]. All patients were younger than 18 years old, had no history of any other tumor, and were diagnosed with glioma based on histopathological examination. Controls were healthy children randomly selected among age and gender-matched children living in the same area as the patients. The Institutional Review Board approved the study of Guangzhou Women and Children's Medical Centre, and all participants' parents or guardians signed an informed consent form.

### 2.2. Genotyping

DNA was extracted from the subjects' blood samples using the QIAamp DNA blood mini kit (QIAGEN, Valencia, CA, USA). *TP53* rs1042522 C>G polymorphism was genotyped by Taqman real-time PCR on a 7900 Sequence Detection System (Applied Biosystems, Foster City, CA). A random selection of 10% of the completed samples was genotyped again to ensure accuracy of genotyping, and the results were 100% consistent with the original genotype.

### 2.3. Statistical Analysis

The two-sided *χ*^2^ test was used to compare the differences in demographic variables and genotype distributions between the case and control groups. A goodness-of-fit test was used to detect deviations from Hardy-Weinberg equilibrium (HWE) for the control group's SNP genotype frequency distribution. To assess the association of the *TP53* rs1042522 C>G polymorphism with glioma risk, we calculated relative odds ratios (ORs) and 95% confidence intervals (CIs) using logistic regression models after adjusting for age and gender. Stratified analysis was performed by age, gender, tumor subtypes, and clinical stages. All statistical analyses were completed using SAS software (version 9.4, SAS Institute, Cary, NC, USA). *P* < 0.05 indicates statistical significance.

## 3. Results

### 3.1. *TP53* rs1042522 C>G Polymorphism and Glioma Risk

We genotyped 171 glioma patients and 228 cancer-free controls successfully. The association between *TP53* rs1042522 C>G polymorphism and glioma risk is shown in Table 1. The genotype frequency distribution in the control group was consistent with Hardy-Weinberg equilibrium (*P* = 0.613). Statistical analysis showed no significant differences in the genotype frequencies between the case and control groups. No correlation was observed between the *TP53* rs1042522 C>G polymorphism and glioma risk, even after adjusting for age and gender.

### 3.2. Stratification Analysis

To further clarify the correlation between *TP53* rs1042522 C>G polymorphism and glioma risk under different stratification conditions, we performed a stratification analysis according to age, gender, tumor subtypes, and clinical stages. The results are presented in Table 2. The results showed no significant association between *TP53* rs1042522 C>G polymorphism and glioma risk.

## 4. Discussion

In this hospital-based case-control study, we explored the association between the *TP53* rs1042522 C>G polymorphism and the risk of childhood glioma. However, we did not detect any significant associations.

The *TP53* gene is a tumor-associated tumor suppressor gene that has received widespread attention. It is located on the short arm of human chromosome 17, and its mutations have been found in various tumors. Mutant *TP53* gene affect tumor proliferation, migration, survival and invasion, and resistance to chemotherapeutic agents [23]. Under physiological conditions, exposure of cells to various stress signals activates the *P53* signaling pathway, causing cells to start multiple transcriptional programs, including cell cycle arrest, DNA repair, senescence, and apoptosis, thereby inhibiting transform of cells [24]. In contrast, mutation-induced *TP53* gene inactivation drives cell invasion, proliferation, and survival, thereby promoting cancer progression and metastasis [25]. Hundreds of polymorphisms in the *TP53* gene are identified to be associated with cancer risk [26]. The rs1042522 C>G polymorphism, the most widely studied polymorphism in *TP53*, has been reported to be related to the risk of various tumors, including breast, prostate, thyroid, bladder, hepatocellular, cervical, osteosarcoma, and neuroblastoma [27–34]. *TP53* rs1042522 C>G is a polymorphism located in exon 72 of exon 4 of the *TP53* gene. The SNP occurs in the structural domain of proline and is a nonsynonymous substitution of C>G. This alteration results in a switch in amino acids from arginine (*Arg*) to proline (*Pro*), which affects the *P53* function and has an impact on the apoptotic process [35]. Mutations in the *TP53* gene are one of the crucial factors contributing to glioma susceptibility [36]. The mutation rate of *TP53* has been reported to be 50-75% in astrocytomas and 10-34% in oligodendrogliomas [37, 38].

A study by Parhar et al. demonstrated that the *TP53* rs1042522 C>G polymorphism might be associated with susceptibility to brain tumors, particularly in high-grade astrocytomas [21]. Malmer et al. revealed that individuals with a family history of cancer are at increased risk of developing brain tumors [39]. In addition, El Hallani et al. found that the *TP53* rs1042522 C>G polymorphism was particularly critical for developing glioblastoma in young patients [40]. In a meta-analysis, the *TP53* rs1042522 C>G polymorphism was found to be negatively associated with the risk of glioblastoma in the overall analysis, as well as stratified analyses by ethnic subgroups, control sources, and glioma subtypes [41]. Another meta-analysis of 2,645 glioma cases and 3,920 control subjects, excluding analysis of glioma subtypes, showed no significant association between the *TP53* rs1042522 C>G polymorphism and risk of glioma [42]. These studies suggest that the effect of *TP53* rs1042522 C>G polymorphism on glioma risk is strongly associated with glioma subtype, genetic factors, and age. In separate analyses based on the association of *TP53* rs1042522 C>G polymorphism with glioma risk in different national populations, *TP53* rs1042522 C>G polymorphism was significantly associated with glioma risk in patients with gliomas in India and China [43–45]. In contrast, no association was found in patients from Portugal and Brazil [46, 47], and these studies mainly focused on predominantly adults. The discrepant results reported by independent research groups suggest that population origin and geography are also important factors influencing the association between *TP53* rs1042522 C>G polymorphism and glioma risk. Therefore, it is essential to elucidate the impact of *TP53* rs1042522 C>G polymorphism on the risk of specific types of tumors in particular populations. In several existing studies, variations in *TP53* have been significantly associated with susceptibility to various pediatric tumors. Of which, *TP53* Pro/Pro genotype was associated with increased susceptibility to acute lymphoblastic leukemia in Caucasian children [48], *TP53* rs1042522 CG/GG genotype had a significantly increased risk of Wilms tumor in children under 18 months of age [49], and *TP53* rs1042522 CG/GG genotype was associated with reduced susceptibility to neuroblastoma [50]. Meanwhile, mutations in *TP53* affect tumor treatment and prognosis. For example, loss-of-function *TP53* mutations predispose children to acute lymphoblastic leukemia and have a significantly higher risk of poor treatment outcomes and even secondary malignancies [51]. In another study, *TP53* codon 72 polymorphism predicted early tumor progression in pediatric hairy cell astrocytoma, guiding the need for aggressive surgical treatment [52]. Few studies have been reported on the association of *TP53* rs1042522 C>G polymorphism with glioma risk in childhood. Our present study mainly explores this area and fills the gap. The current study did not detect any association between *TP53* rs1042522 C>G polymorphism and glioma risk in children, even after stratified study population with age, gender, tumor subtypes, and clinical stages. It should be noted that more studies are needed to confirm our observations due to multiple factors limiting our study. Firstly, our study's relatively small sample size and the low epistatic susceptibility of individual polymorphisms may weaken the statistical power. Secondly, the interaction between various genetic polymorphisms during tumorigenesis, and the fact that we focused on only one polymorphism, may lead to false-negative results. Thirdly, selection bias may be unavoidable because our study was hospital-based. Fourth, environmental factors and environmental-genetic interactions contributed to glioma, but these factors were not investigated in the risk model.

Despite these limitations, the present study is the most extensive study to date on the association of *TP53* rs1042522 C>G polymorphism with glioma risk in Chinese children, and further studies on the susceptibility of *TP53* rs1042522 C>G polymorphism may provide new insights into the etiology of glioma, which could be necessary for providing more appropriate treatment for specific populations.

## 5. Conclusions

In summary, the *TP53* rs1042522 C>G polymorphism may not be significantly associated with glioma risk in Chinese children. However, a more extensive sample size study should be conducted to validate our results.

## Figures and Tables

**Table 1:** Association between *TP53* rs1042522 C>G polymorphism and glioma risk.

Genotype	Cases (*N* = 171)	Controls (*N* = 228)	*P* ^a^	Crude OR (95% CI)	*P*	Adjusted OR (95% CI)^b^	*P* ^b^
rs1042522 (HWE = 0.613)							
CC	54 (31.58)	65 (28.51)		1.00		1.00	
CG	80 (46.78)	117 (51.32)		0.82 (0.52-1.30)	0.406	0.83 (0.52-1.32)	0.433
GG	37 (21.64)	46 (20.18)		0.97 (0.55-1.70)	0.911	1.01 (0.57-1.78)	0.981
Additive			0.822	0.97 (0.73-1.28)	0.822	0.99 (0.74-1.31)	0.922
Dominant	117 (68.42)	163 (71.49)	0.507	0.86 (0.56-1.33)	0.507	0.88 (0.57-1.36)	0.564
Recessive	134 (78.36)	182 (79.82)	0.722	1.09 (0.67-1.78)	0.721	1.13 (0.69-1.85)	0.630

OR: odds ratio; CI: confidence interval; HWE: Hardy-Weinberg equilibrium. ^a^*χ*^2^ test for genotype distributions between glioma patients and cancer-free controls. ^b^Adjusted for age and gender.

**Table 2:** Stratification analysis between *TP53* rs1042522 C>G polymorphism and glioma risk.

Variables	rs1042522 (cases/controls)		Crude OR	*P*	Adjusted OR^a^	*P* ^a^
	CC	CG/GG	(95% CI)		(95% CI)	
Age, month						
<60	29/32	56/87	0.71 (0.39-1.30)	0.267	0.71 (0.39-1.30)	0.269
≥60	25/33	61/76	1.06 (0.57-1.97)	0.855	1.08 (0.58-2.01)	0.814
Gender						
Female	27/27	54/66	0.82 (0.43-1.56)	0.541	0.81 (0.42-1.56)	0.534
Male	27/38	63/97	0.91 (0.51-1.64)	0.764	0.93 (0.52-1.68)	0.815
Subtypes						
Astrocytic tumors	39/65	86/163	0.88 (0.55-1.41)	0.596	0.91 (0.56-1.48)	0.709
Ependymoma	9/65	16/163	0.71 (0.30-1.69)	0.436	0.69 (0.29-1.65)	0.405
Neuronal and mixed	3/65	11/163	1.46 (0.40-5.41)	0.569	1.37 (0.37-5.14)	0.638
Embryonal tumors	3/65	4/163	0.53 (0.12-2.44)	0.417	0.67 (0.12-3.69)	0.647
Clinical stages						
I	31/65	72/163	0.93 (0.56-1.54)	0.768	0.92 (0.55-1.55)	0.764
II	9/65	19/163	0.84 (0.36-1.96)	0.689	0.84 (0.36-1.96)	0.684
III	6/65	9/163	0.60 (0.21-1.75)	0.348	0.57 (0.19-1.67)	0.302
IV	8/65	17/163	0.85 (0.35-2.06)	0.715	1.21 (0.45-3.21)	0.706
I+II	40/65	91/163	0.91 (0.57-1.45)	0.685	0.91 (0.56-1.46)	0.684
III+IV	14/65	26/163	0.74 (0.36-1.51)	0.407	0.83 (0.40-1.73)	0.623

OR: odds ratio; CI: confidence interval. ^a^Adjusted for age and gender: omitting the corresponding stratify factor.

## Data Availability

All the data were available upon request.

## Abbreviations

CNS: Central nervous system
GBM: Glioblastoma
GWAS: Genome-wide association study
SNP: Single-nucleotide polymorphism
OR: Odds ratio
CI: Confidence interval
HWE: Hardy-Weinberg equilibrium.

## Supplementary Materials

Supplementary Materials Table S1: frequency distribution of selected variables in glioma patients and cancer-free controls.
