# Supplementary material for: Association of TP53 rs1042522 C>G Polymorphism with Glioma Risk in Chinese Children
Source: Biomed Res Int. 2022 Aug 13;2022:2712808. doi: 10.1155/2022/2712808 (PMC9392611; doi:10.1155/2022/2712808)
Supplement: Supplementary Materials — Table S1: frequency distribution of selected variables in glioma patients and cancer-free controls. [file 2712808.f1.doc]

| **Table S1**. Frequency distribution of selected variables in glioma patients and cancer-free controls | | | | | |
| --- | --- | --- | --- | --- | --- |
| Variables | Cases (N=171) | | Controls (N=228) | | *P* ^a^ |
|  | No. | % | No. | % |  |
| Age range, month | 4.00-168.00 | | 4.00-168.00 | | 0.623 |
| Mean ± SD | 63.40 ± 47.72 | | 52.41 ± 32.65 | |  |
| <60 | 85 | 49.71 | 119 | 52.19 |  |
| ≥60 | 86 | 50.29 | 109 | 47.81 |  |
| Gender |  |  |  |  | 0.190 |
| Female | 81 | 47.37 | 93 | 40.79 |  |
| Male | 90 | 52.63 | 135 | 59.21 |  |
| Subtypes | | | | | |
| Astrocytic tumors | 125 | 73.10 | / | / |  |
| Ependymoma | 24 | 14.62 | / | / |  |
| Neuronal and mixed neuronal-glial tumors | 14 | 8.19 | / | / |  |
| Embryonal tumors | 7 | 4.09 | / | / |  |
| WHO stages | | | | | |
| I | 103 | 60.23 | / | / |  |
| II | 28 | 16.37 | / | / |  |
| III | 15 | 8.77 | / | / |  |
| IV | 25 | 14.62 | / | / |  |
| SD, standard deviation.  ^a^ Two-sided χ^2^ test for distributions between glioma patients and cancer-free controls. | | | | | |
